# Repair of a type B aortic dissection with a re-vascularization of the aberrant right subclavian artery in an adult patient

**DOI:** 10.1186/s13019-019-1031-7

**Published:** 2019-11-27

**Authors:** Mahmoud Yousef Ibrahim Abuharb, Bian Xiao Ming, He Jian

**Affiliations:** 1 Department of Cardiothoracic Surgery, First Affiliated Hospital of Dalian Medical University, Lianhe Avenue, Dalian, China; 2 Department of Cardiothoracic Surgery, First Affiliated Hospital of Dalian Medical University, Zhongshan Avenue 222, Dalian, China

**Keywords:** Aberrant, Lusoria, Stanford B, Dissection, Elephant trunk

## Abstract

**Background:**

An aberrant right subclavian artery which arises from the proximal descending aorta may result in aortic dissection. The dissection may occur at either the site of the primary intimal tear or from an aortic branch. These conditions may lead to blood flow limitation and possible aneurysmal degeneration in the future.

**Case presentation:**

We described the clinical presentation and management of a 54-year old patient diagnosed with a rare case of an aberrant right subclavian artery with Stanford Type B aortic dissection. A hybrid surgical approach was successfully performed and the patient had an uneventful recovery.

**Conclusion:**

Even though aortic dissection is often an incidental finding, this case highlighted that in rare situations, it can be associated with an aberrant right subclavian artery. It is important to disseminate this association as it has profound diagnostic and therapeutic implications in safeguarding the clinical outcomes of patients with such condition.

## Background

Among the various types of anatomic variations associated with the aortic arch, an aberrant right subclavian artery (ARSA) is the most frequently encountered. The incidence of ARSA is reported as between 0.5 and 2.0% in the general population [1]**.** As high as 60% of the ARSA patients may suffer from aneurysmal dilatation that may lead to Kommerell’s diverticulum (KD), a condition frequently associated with an increased risk of rupture and dissection [2]**.**

In contrast, there are very few reported cases of ARSA patients complicated with acute type B aortic dissection who were not diagnosed with a KD. Our patient in this case report was one of such rare cases. Most patients with this condition are asymptomatic and do not present with any obvious clinical symptoms. Thus, the condition is often an incidental finding.

To date, the reported incidence of ARSA with an associated acute type B aortic dissection is very low. Unreported variations like these often pose difficult challenges for the surgeons in their attempts to repair any ruptures. As a result, novel surgical approaches have been established to manage this condition, including thoracic endovascular aortic repair (TEVAR), TEVAR with the chimney method, hybrid method, and frozen elephant trunk**.** In this case report, we described a rare case of ARSA with acute type B aortic dissection. We also outlined the hybrid surgical method using the frozen elephant trunk procedure in the repair of the anomaly.

### Case presentation

**Fig. 1:**
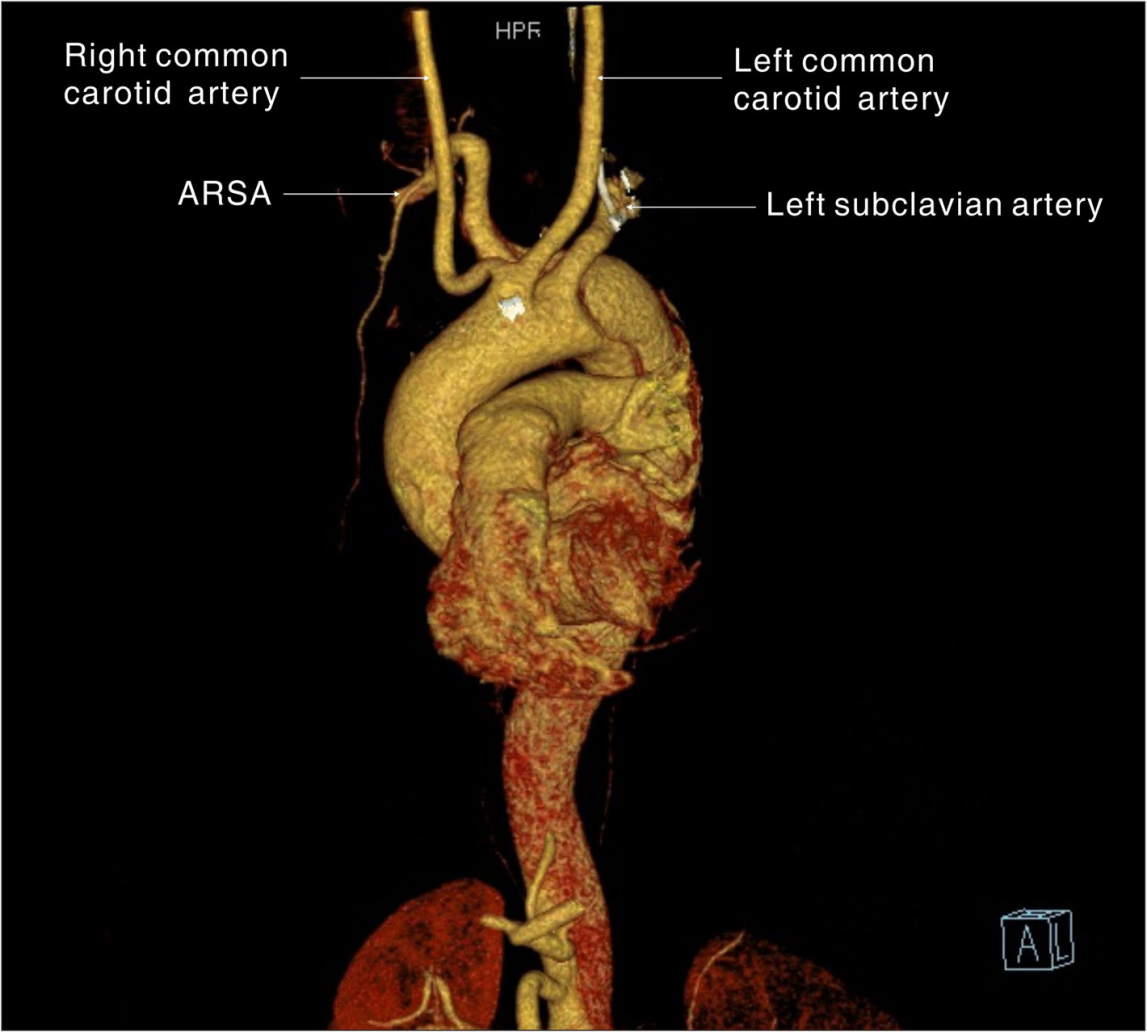
A 3D computed tomography reconstruction shows the aberrant right subclavian artery running distally to the left subclavian artery

**Fig. 2:**
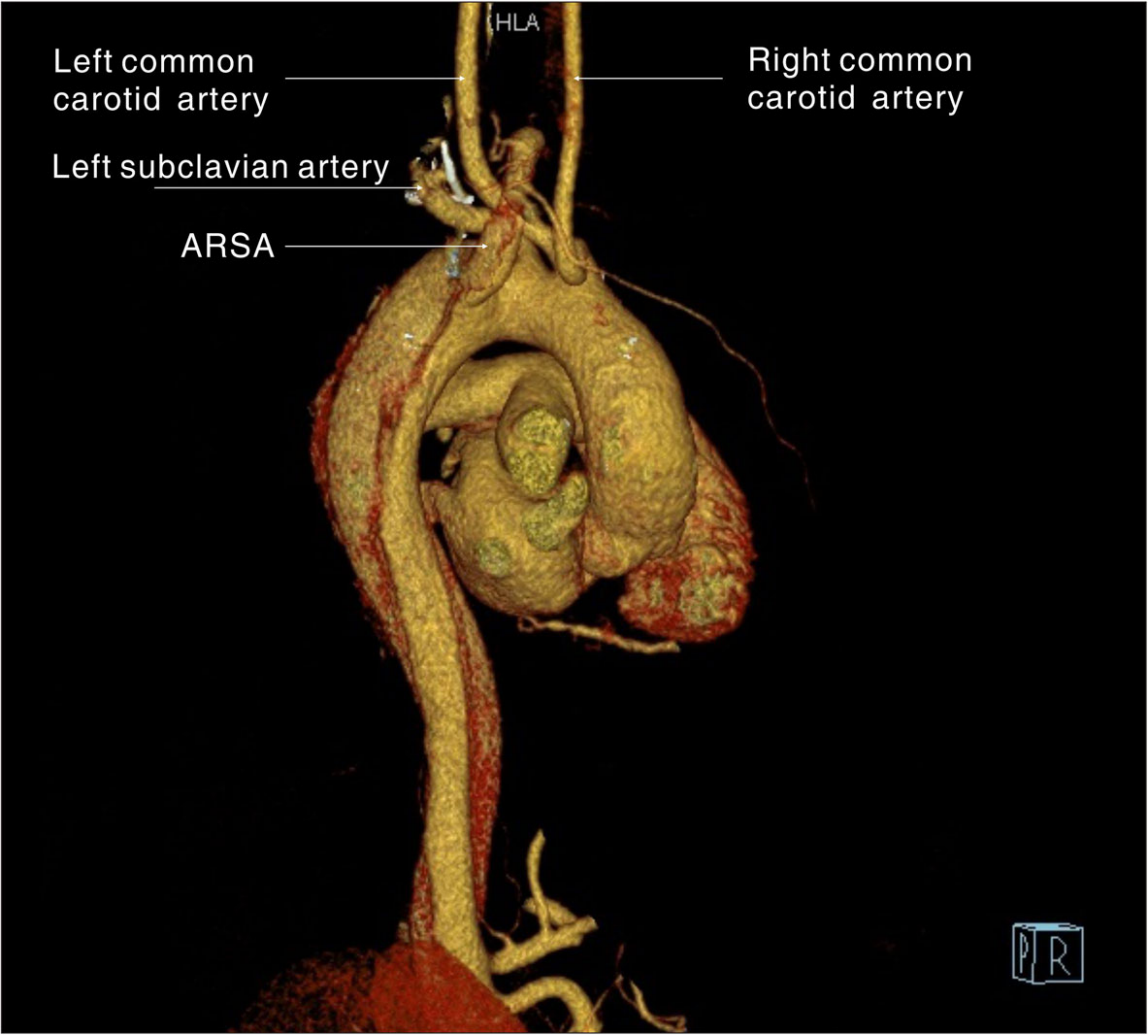
3D computed tomography reconstruction shows the aberrant right subclavian artery running distally to the left subclavian artery

**Fig. 3:**
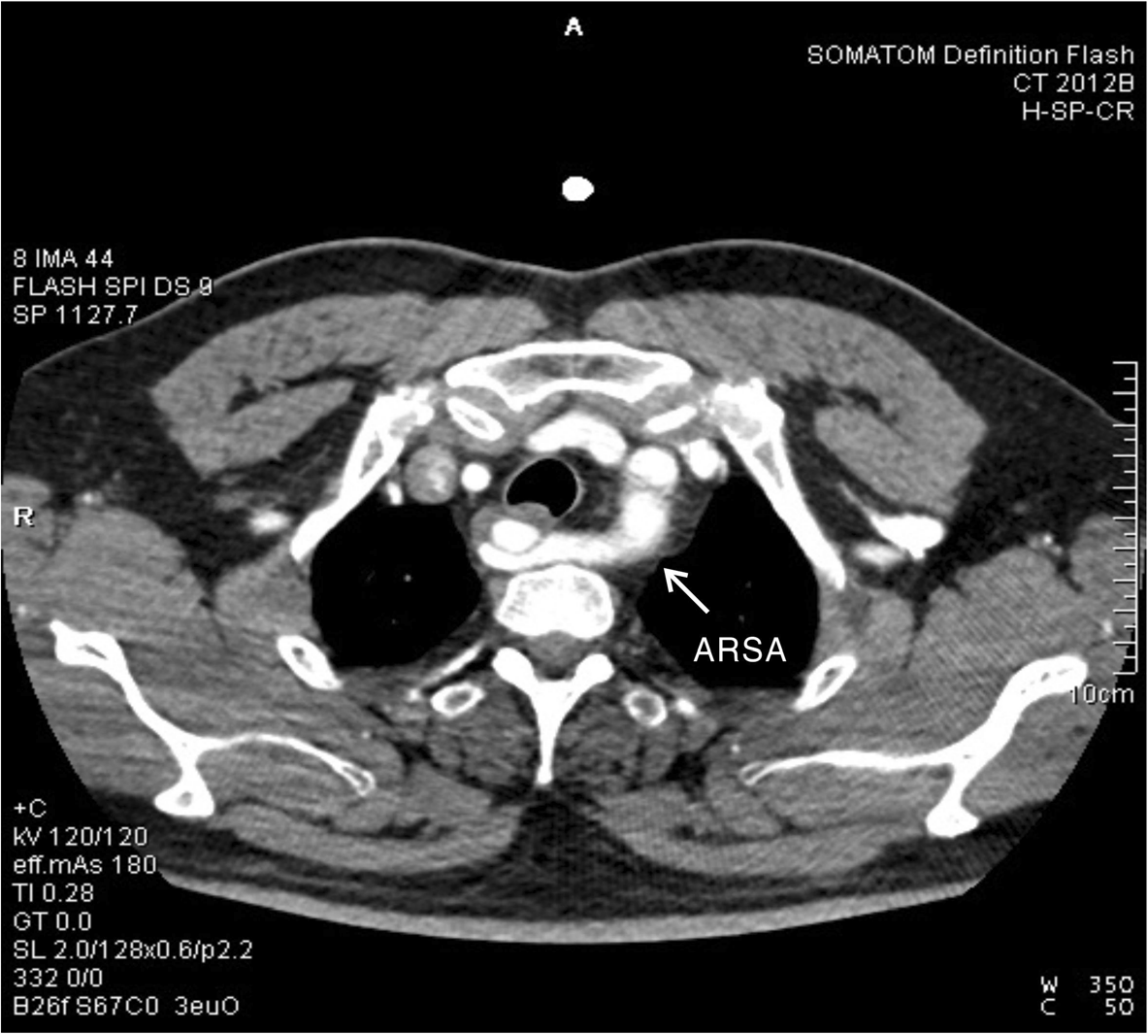
Computed tomography shows a Stanford type B aortic dissection involving the aberrant right subclavian artery

A 54-year old male patient was admitted to the hospital with a sudden onset of chest pain and backache for 8 h. Physical examination showed blood pressure (BP) of 126/73 mmHg on the upper right arm, 136/69 mmHg on the upper left arm, 158/77 mmHg on the lower right limb, and 146/70 mmHg on the lower left limb. Heart rate was 110 times/min with normal breath sounds in both lungs. No murmur heard upon auscultation. His abdomen was soft and non-tender. There was no edema in both lower limbs. Electrocardiogram (ECG) showed a sinus rhythm of 101 beats/min. Echocardiography (ECHO) showed that the left atrium was 42 × 54 mm, the right atrium was 38 × 48 mm, the right ventricle was 18 mm, the pulmonary artery was 23 mm, and the left ventricle was 53 mm with a left ventricular ejection fraction (LVEF) of 56%. The findings suggested ventricular septal thickening with mild to moderate insufficiency over the left atrial aortic valve. Arterial blood gas analysis showed PaO2 40.8 mmHg, PaCO2 64.0 mmHg, SpO2 92.8%, and Hgb 13.4 g/L. Aortic computed tomography angiography (CTA) confirmed a Stanford type B aortic dissection with an aberrant right subclavian artery (Fig. 1, 2, and 3) and the aortic diameter was 6 cm.

### Surgical procedure

**Fig. 4:**
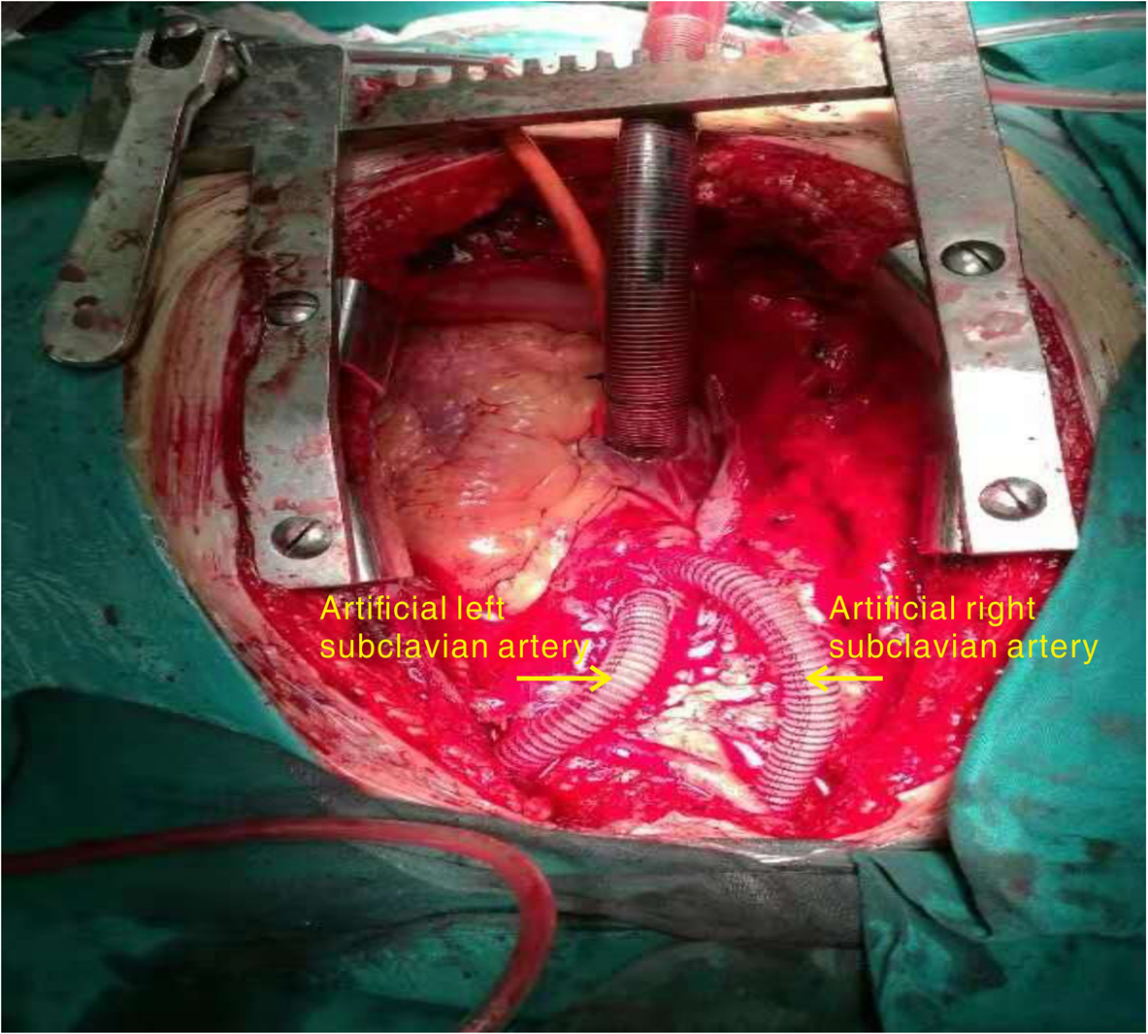
Simple computer sketching shows the frozen elephant trunk procedure used in the repair

After admission, the patient was started on analgesics and started on anti-hypertensive to control the blood pressure. Two days after initial admission, the patient underwent surgical repair via a median sternotomy approach. Frozen elephant trunk hybrid procedure was employed (Fig. 4). An incision was made on the anterior wall of the aortic arch to implant the surgical graft onto the distal aorta. By using the stented elephant trunk, the origin of the ARSA was blocked.

**Fig. 5:**
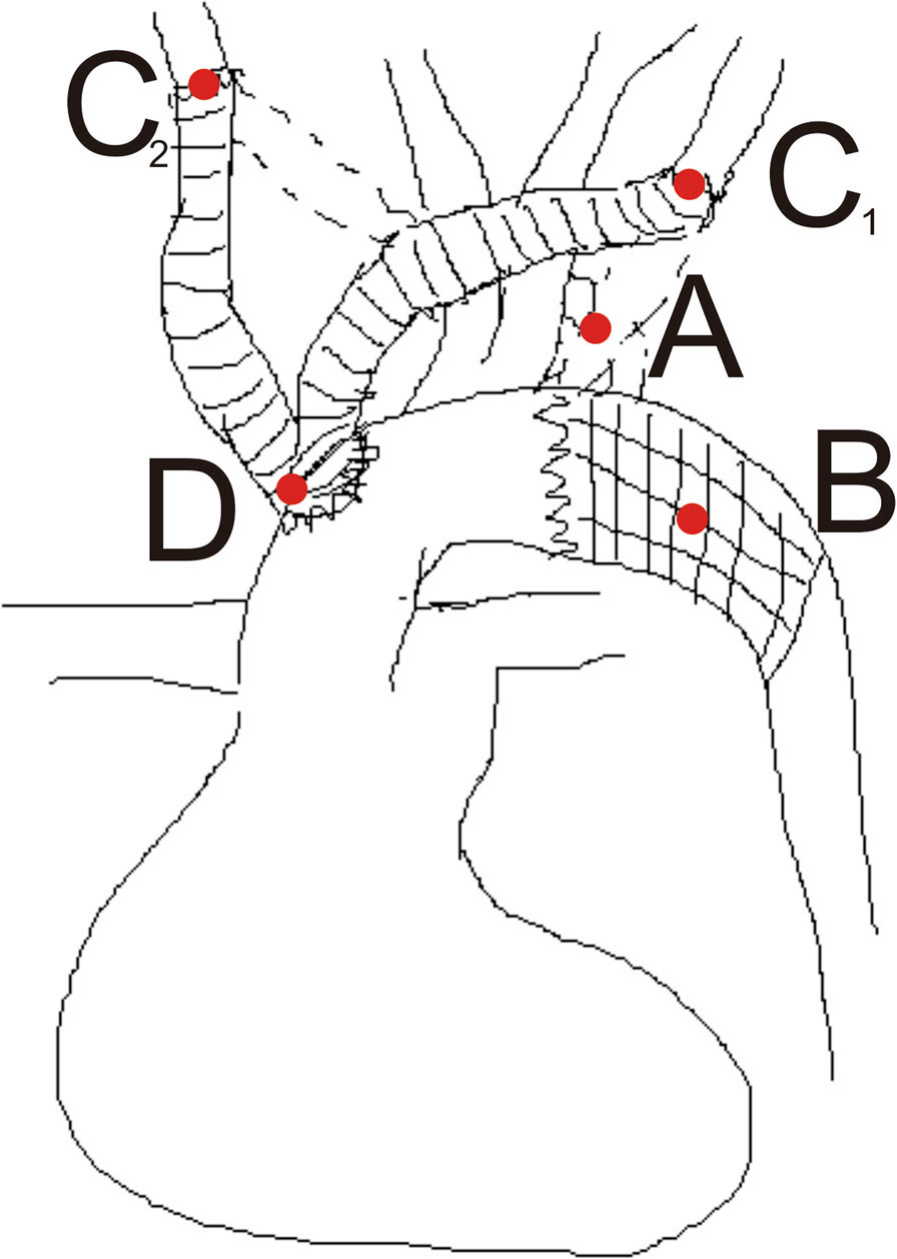
Intra-operative image shows the artificial blood vessels used as bypass in the left subclavian artery and the aberrant right subclavian artery

As for the distal aorta, the section containing the surgical graft was anastomosed distally to the normal segment of the proximal normal aortic segment with a firm suture. Next, a bypass of the ARSA and left subclavian artery was created to the new location on the ascending aorta (Fig. 5).

The patient was extubated 1 day after the surgical procedure. He was discharged from hospital on day 13 post operation. He had no chest pain post-operatively. His activity tolerance also improved. The patient returned for medical check-up after 1 month and he was well with no active complaints.

## Discussion

There are many types of congenital cardiac anomalies, among which ARSA is the most frequently type of aortic arch anomalies. ARSA is often diagnosed as an incidental radiological finding due to its asymptomatic nature. However, in certain rare cases, it may manifest as compressive symptoms on the trachea and esophagus. In the pediatric age group, dyspnea is a common presenting symptom as the trachea in infants are compressible. Therefore, pediatric patients would present with the typical signs and symptoms of compression which are respiratory in nature, such as wheezing, stridor, recurrent pneumonia, and cyanosis. A few cases had been reported about pediatric patients with KD and tracheal compression who presented with symptoms that mimicked asthma.

On the contrary, respiratory symptoms are rare among adult patients. ARSA in adults often leads to esophageal compression. Thus, dysphagia to solid remains the most common presenting complaint among majority of the adult patients with ARS [3, 4]. However, our patient was completely asymptomatic and complained of neither dysphagia nor dyspnea.

In terms of diagnostic modality for ARSA patients, an esophagogram may be the initial test of choice. However, computed tomography (CT) or magnetic resonance imaging (MRI) remains the gold standard of diagnosis. Aortic CTA is the preferred investigation modality as it has a high diagnostic sensitivity and specificity for ARSA. In the interest of time and cost, we performed CTA directly for the patient. Apart from identifying the exact location and extent of ARSA, CTA is able to measure the inner diameter of the aortic dissection and the thickness of the wall in an accurate manner. This would be important to detect any pericardial and pleural effusion, mediastinal hematoma, or aortic pseudoaneurysm.

To date, ARSA complicated by type B aortic dissection is rarely reported in the literature [5]. As a result, no standard surgical approach has been established for the management of this condition. Certain asymptomatic patients are managed conservatively. However, surgical repair is warranted for patients with respiratory distress or dysphagia. In view of the persistent pain, threatened exsanguination, mal-perfusion (renal and limbs), rapid aortic enlargement, and uncontrolled hypertension in our patient, surgical repair was indicated. With an underlying ARSA, the surgical management of type B dissection represents a high level of technical difficulty and risks. Initially, TEVAR was considered as the surgical option. However, we decided against it because of the unfavorable anatomy of the underlying lesion, which included inadequate proximal and distal seal zones, tortuosity, lack of vascular access options, and extremes of aortic diameter. Therefore, open surgery was deemed as a better approach.

In the conventional approach of the open surgery for the repair of this anomaly, replacement of the affected aorta proximal to the origin of the ARSA would be performed with preservation of the ARSA. After revascularization, the diseased aortic lesion would be repaired. However, in this case, we had to adopt a novel surgical approach. Based on the imaging findings, the patient had a Stanford type B aortic dissection with a rupture in the descending aorta. The site of rupture was less than 1 cm from the left subclavian artery. Thus, it was located very closely between the right and left subclavian arteries, making it a highly complicated surgical procedure. Therefore, a novel alternative surgical repair method was devised.

During the operation, the openings to the right and left subclavian arteries were found to be obstructed after we employed the stent graft. As a result, a bypass was created to the ascending aorta. Proximal and distal anastomoses were established using artificial blood vessels. The artificial blood vessels were modelled as an ‘island-shape’ to directly match the aorta. This was able to reduce the anastomosis time and the risk of bleeding. In view of the high risk of embolization, cannulation of the carotid artery was performed and close attention was paid to brain protection throughout the operation. No brain complications were detected post-operatively.

## Conclusion

Low incidence of reporting on the rare cases of ARSA with an associated acute type B aortic dissection can pose difficult challenges in the surgical repair. This case report outlined the condition and the frozen elephant trunk method used to repair the anomaly. We believe that it is a safe, effective, and convenient method as it is can be performed as a single stage procedure to allow the repair of any concomitant lesions in the heart including at the proximal aorta and aortic arch.

## Data Availability

All data are available upon request from the Department of Cardiothoracic Surgery at the First Affiliated Hospital of Dalian Medical University.

## Abbreviations

ARSA: Aberrant right subclavian artery
CTA: Computed tomography angiography
ECG: Electrocardiogram
ECHO: Echocardiography
KD: Kommerell diverticulum
LVEF: Left ventricular ejection fraction
MRI: Magnetic resonance imaging
TEVAR: Thoracic endovascular aortic repair
